# Supplementary material for: Antinociceptive Potential of Ximenia americana L. Bark Extract and Caffeic Acid: Insights into Pain Modulation Pathways
Source: Pharmaceuticals (Basel). 2024 Dec 11;17(12):1671. doi: 10.3390/ph17121671 (PMC11677608; doi:10.3390/ph17121671)
Supplement: Supplementary file 1 [file pharmaceuticals-17-01671-s001.zip › pharmaceuticals-3329185-supplementary.pdf]

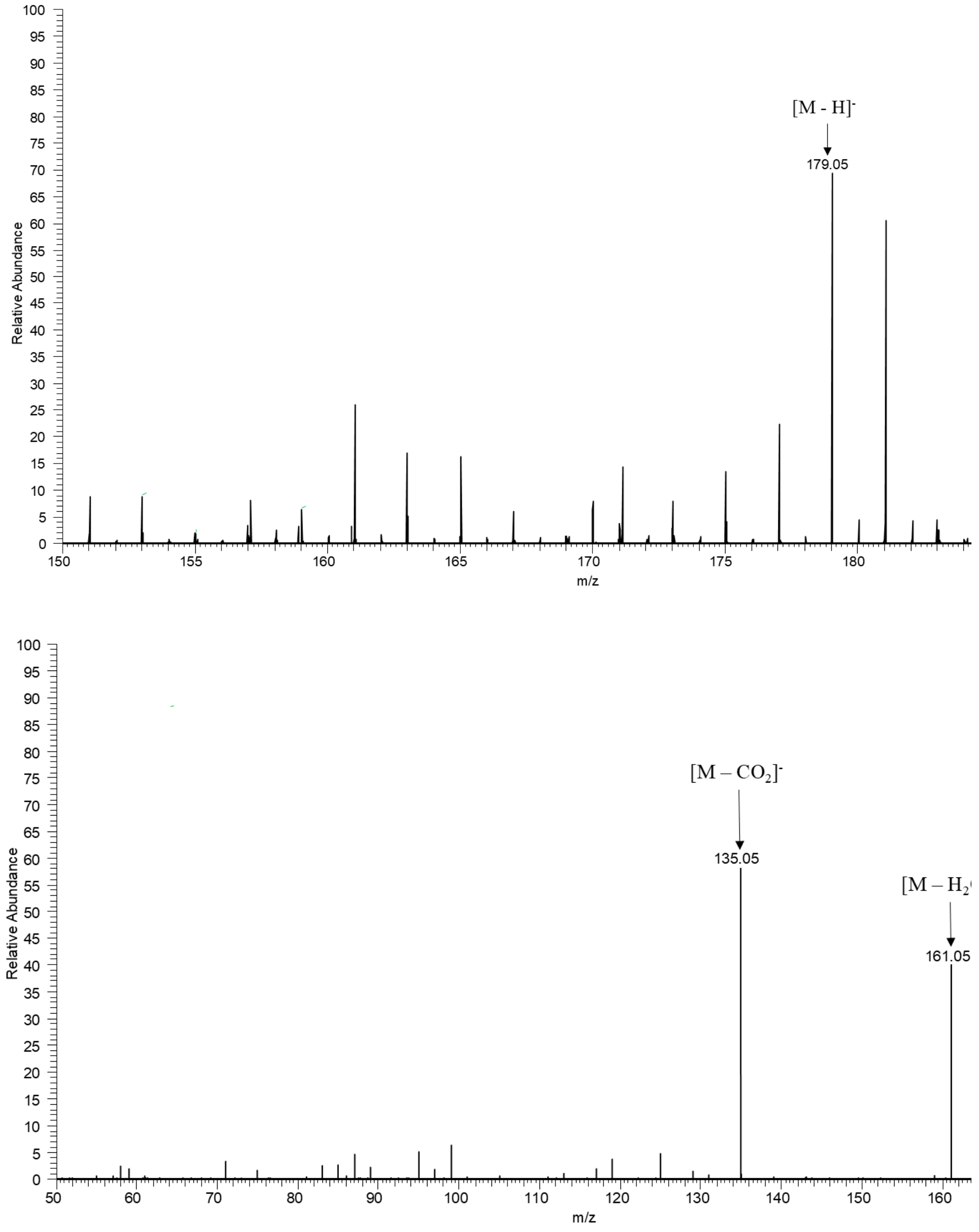

Figure S1. Chromatogram of ESI-MS scanning by UPLC-MS. **1A.** ESI-MS scanning spectrum in negative ionization mode for the range of *m/z* 150-190. It is possible to observe the peak at m/z 179.05 referring to the caffeic acid product. **1B.** ESI-MS/MS full scan spectrum in negative ionization mode, relevant to the peak at *m/z* 179.05 observed in the MS spectrum for the caffeic acid product.

Table S1: Results of compound annotation in the hydroethanolic extract of *X. americana* L. through molecular network analysis.

Parent mass	Adduct	Molecular formula	Metabolite name	Chemical structure	Chemical class
169,014	$[M-H]^-$	$C_7H_6O_5$	Gallic acid	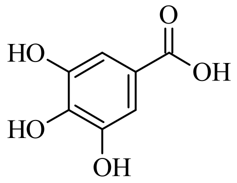	Organic acid
179,034	$[M-H]^-$	$C_9H_8O_4$	Caffeic acid	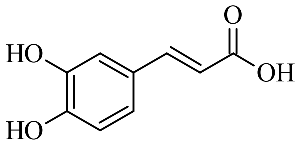	Organic acid
195,050	$[M-H]^-$	$C_6H_{12}O_7$	Gluconic acid	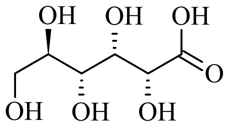	Organic acid
193,034	$[M-H]^-$	$C_6H_{10}O_7$	D-Glucuronic acid	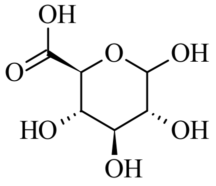	Organic acid
285,039	$[M-H]^-$	$C_{15}H_{10}O_6$	Kaempferol	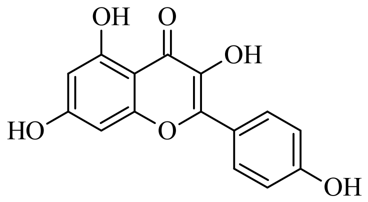	Flavonoid
289,071	$[M-H]^-$	$C_{15}H_{14}O_6$	(-)-Catechin	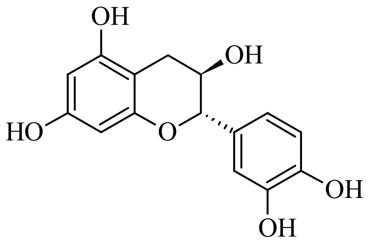	Flavonoid
301,035	$[M-H]^-$	$C_{15}H_{10}O_7$	Quercetin	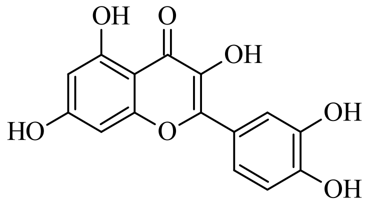	Flavonoid

609,147	[M-H]$^-$	$C_{27}H_{30}O_{16}$	Rutin	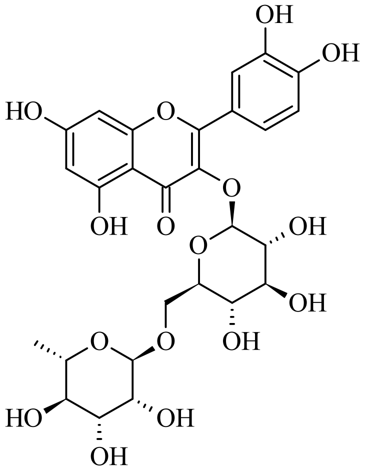	Flavonoid
